# hPMSCs protects against d-galactose-induced oxidative damage of CD4^+^ T cells through activating Akt-mediated Nrf2 antioxidant signaling

**DOI:** 10.1186/s13287-020-01993-0

**Published:** 2020-11-04

**Authors:** Yanlian Xiong, Yueming Wang, Jiashen Zhang, Nannan Zhao, Hengchao Zhang, Aiping Zhang, Dongmei Zhao, Zhenhai Yu, Yancun Yin, Lele Song, Yanlei Xiong, Xiying Luan

**Affiliations:** 1 Department of Anatomy, School of Basic Medicine, Binzhou Medical University, Yantai, People’s Republic of China; 2 Department of Immunology, School of Basic Medicine, Binzhou Medical University, Yantai, People’s Republic of China; 3 Department of Pathology, Xuanwu Hospital, Capital Medical University, Beijing, People’s Republic of China

**Keywords:** Oxidative stress, Aging, Senescence-associated secretoryphenotype, Nrf2, CD4^+^ T cells, hPMSC

## Abstract

**Background:**

Mesenchymal stem cells (MSCs) were considered a regenerative therapeutic approach in both acute and chronic diseases. However, whether MSCs regulate the antioxidant metabolism of CD4^+^ T cells and weaken immunosenescence remains unclear. Here, we reported the protective effects of hPMSCs in aging-related CD4^+^ T cell senescence and identified the underlying mechanisms using a d-gal-induced mouse aging model.

**Methods:**

In vivo study, 40 male C57BL/6 mice (8 weeks) were randomly divided into four groups: control group, d-gal group, hPMSC group, and PBS group. In in vitro experiment, human naive CD4^+^ T (CD4CD45RA) cells were prepared using a naive CD4^+^ T cell isolation kit II and pretreated with the Akt inhibitor LY294002 and Nrf2 inhibitor ML385. Then, isolated naive CD4^+^ T cell were co-cultured with hPMSCs for 72 h in the absence or presence of anti-CD3/CD28 Dynabeads and IL-2 as a mitogenic stimulus. Intracellular ROS changes were detected by flow cytometry. The activities of the antioxidant enzymes superoxide dismutase, glutathione peroxidase, and catalase were measured by colorimetric analysis. The senescent T cells were detected SA-β-gal stain. The expression of aging-related proteins was detected by Western blotting, RT-PCR, and confocal microscopy.

**Results:**

We found that hPMSC treatment markedly decreased the ROS level, SA-β-gal-positive cells number, senescence-associated secretory phenotype (IL-6 and OPN) expression, and aging-related protein (P16 and P21) expression in senescent CD4^+^ T cells. Furthermore, hPMSC treatment effectively upregulated Nrf2 nuclear translocation and the expression of downstream target genes (HO-1, CAT, GCLC, and NQO1) in senescent CD4^+^ T cells. Moreover, in vitro studies revealed that hPMSCs attenuated CD4^+^ T cell senescence by upregulating the Akt/GSK-3β/Fyn pathway to activate Nrf2 functions. Conversely, the antioxidant effects of hPMSCs were blocked by the Akt inhibitor LY294002 and Nrf2 inhibitor ML385 in senescent CD4^+^ T cells.

**Conclusions:**

Our results indicate that hPMSCs attenuate d-gal-induced CD4^+^ T cell senescence by activating Nrf2-mediated antioxidant defenses and that upregulation of Nrf2 by hPMSCs is regulated via the Akt/GSK-3β/Fyn pathway.

## Introduction

With the aging process, the immune system function gradually declines, leading to alterations in innate and adaptive immunity in older individuals, which is designated “immunosenescence” [1]. Immunosenescence affects function and compartment of T cells, leading to age-related immune function decline and increases the susceptibility of elderly individuals to cancers and infectious diseases [2]. It is evident that aging decline alterations in CD4^+^ T cell immunity was accompanied by aging [3]. CD4^+^ T cell senescence is characterized by oncogene, reactive oxygen species (ROS) activation, or tumor suppressor genes inactivation, leading to irreversible proliferation arrest [4, 5]. Therefore, improving the antioxidant capacity of CD4^+^ T cells and reducing oxidative stress damage may delay the process of immune aging.

In aging, increasing oxidative stress was induced by various metabolites, on the contrary, as the primary lines of defense, the activity of antioxidant enzymes decreased. Nuclear factor erythroid-2-related factor 2 (Nrf2) is a crucial regulator of the cellular antioxidant system, which regulates the expression of a variety of key antioxidant enzymes [6–8]. There is evidence that activated Nrf2 protection against the phenotypic changes and mitochondrial function in memory T cells, relieve aged-related oxidant injury [9]. Increasing evidence suggests that Nrf2 pathway is essential in regulating the innate immune system function [10]. Together, these data suggest that interfering with Nrf2 antioxidant signal provides a rational approach to alleviate cellular immune dysfunction during aging.

Mesenchymal stem (or stromal) cells (MSCs) was considered a regenerative therapeutic approach in both acute and chronic diseases [11, 12]. Recently, it has been found that MSCs possess potent immunomodulatory and anti-inflammatory properties [13–15]. Interestingly, a recent study indicated that treatment with MSCs increased Nrf2 expression and activated the downstream antioxidant HO-1, leading to inhibition of oxidative stress, cell apoptosis and the inflammatory response in lung tissue [16]. However, whether MSCs regulate the antioxidant metabolism in senescent T cells via Nrf2-mediated exogenous antioxidant defenses and its influence on aging-related T cell dysfunction remain to be elucidated.

Our findings provided evidence that supports an important role for hPMSCs in attenuating d-galactose (d-gal)-triggered CD4^+^ T cell senescence by activating Nrf2-mediated exogenous antioxidant defenses and revealed the protective effect of hPMSCs in reducing oxidative damage in senescent CD4^+^ T cells, thereby clinically alleviating immunosenescence.

## Materials and methods

### Reagents

RNA extraction kit was from Qiagen (Qiagen Inc., CA, USA). DAPI (#4083) and antibodies against Nrf2 (#12721), Akt (#9272), HO-1 (#43966), CAT (#14097), GSK-3β (#12456), NQO1 (#62262), P16 (#80772), P21 (#2947), p-Akt (#4060), p-GSK-3β (#5558), Fyn (#4023), Histon H3 (#4499), and β-actin (#4970) were obtained from Cell Signalling Technology Corporation (MA, USA). CD4^+^ T Cell Isolation Kit II was obtained from Miltenyi (Bergisch Gladbach, Germany). SA-β-Gal staining kit was obtained from Beyotime (Shanghai, China). LY294002 (L9908) and ML385 (SML1833) were obtained from Sigma-Aldrich (St. Louis., MO, USA).

### Animal models

Male C57BL/6 mice (8 weeks) were obtained from Binzhou Medical University (Yantai, China). Mice were housed in a standard environment with a regular light/dark cycle and free access to water and chow diet. All experimental procedures were approved by the Binzhou Medical University Institutional Animal Care and Use Committee and this study was conducted in accordance with the National Laboratory Animal Care and Use research committee guidelines (permit number, 2018-05).

**Fig. 1:**
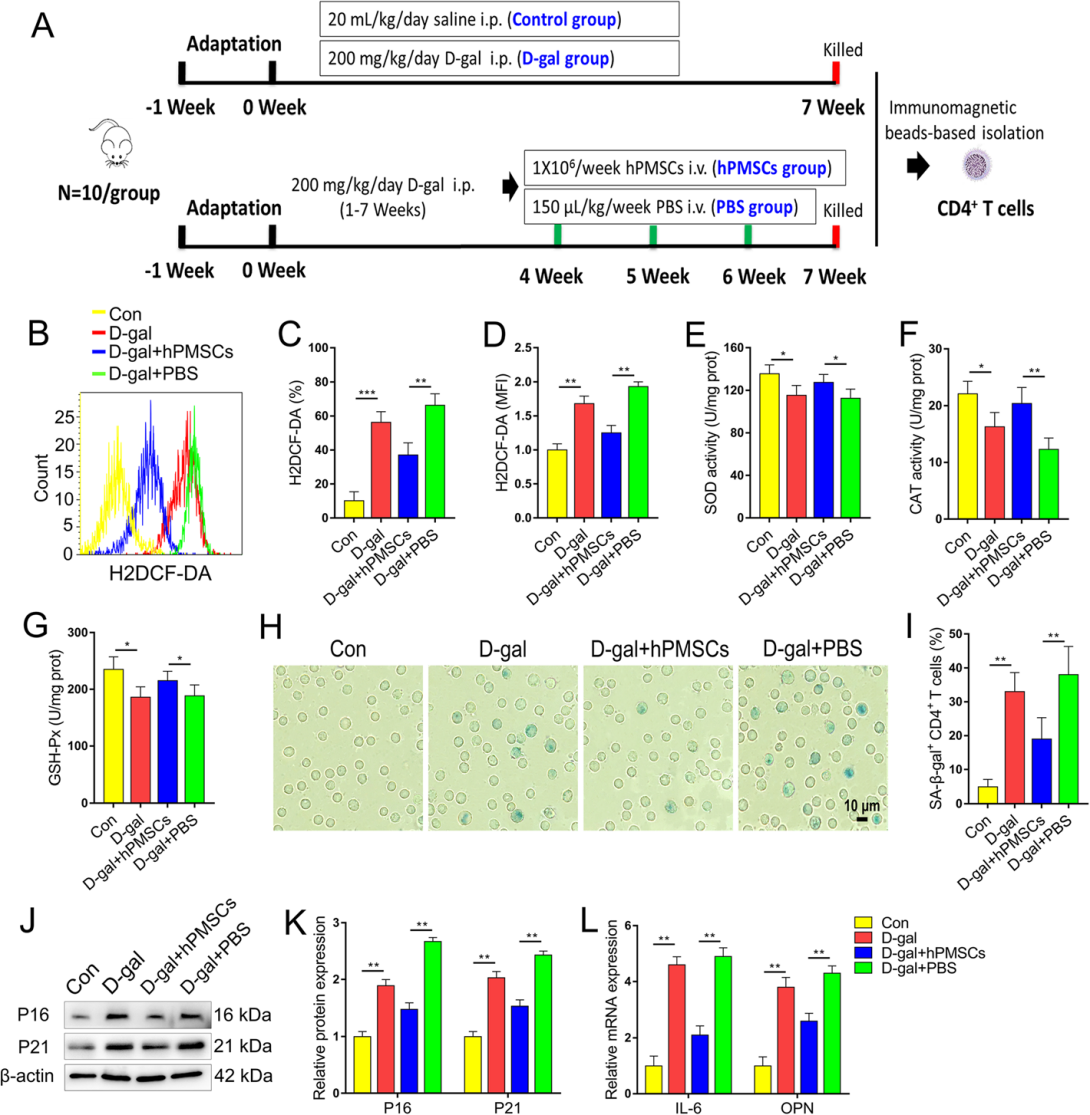
hPMSCs relieves d-gal-triggered CD4^+^ T cells senescence in mice. **a** The scheme of the experimental design. **b**–**d** The expression of ROS in CD4^+^ T cells. **e**–**g** The antioxidant enzyme activity of SOD, CAT, and GSH-Px in CD4^+^ T cells. **h**, **i** The percentages of SA-β-gal-positive CD4^+^ T cells are shown (bar = 10 μm). **j**, **k** The expressions of senescent markers P16 and P21 in CD4^+^ T cells. **l** The mRNA expressions of IL-6 and OPN in CD4^+^ T cells. Data represent the mean scores ± SEM of at least three independent experiments. *p < 0.05, **p < 0.01 (*n* = 10 animals per group)

The mice were randomly divided into four groups (*n* = 10 animals per group): (1) control group, treated with saline (20 mL kg^− 1^ day^− 1^) as a vehicle for 7 weeks; (2) d-gal group, treated with d-gal (200 mg kg^− 1^ day^− 1^) for 7 weeks; and (3) mice were treated with d-gal (200 mg kg^− 1^ day^− 1^) for 7 weeks, followed by 1 × 10^6^ hPMSCs week^− 1^, or (4) 150 μL PBS kg^− 1^ week^− 1^ intravenously on the first day of weeks 5–7 (hPMSC group and PBS group, respectively). hPMSCs (1 × 10^6^ hPMSCs precipitate in 150 μL PBS per mice) or an equal volume of PBS (150 μL) were administered intravenously over a period of 2 min via the tail vein. d-gal and saline were administered intraperitoneally. Detailed animal experimental designs are presented in Fig. 1a. CD4^+^ T cells were isolated from single-cell suspensions of splenocytes using a magnetic cell separator as described previously [17].

### Isolation of hPMSCs

hPMSCs were isolated from human term placentas of donors as described previously [18]. The hPMSCs isolation procedure was approved by the Research Ethics Committee of the Yantai Yuhuangding Hospital. Isolated hPMSCs were identified by detection of cell morphology using microscopy and cell surface antigens CD34, CD105, CD90, CD19, CD73, CD14, and HLA-DR using flow cytometry (FCM). The FCM results indicated that more than 95% of isolated hPMSCs expressed CD73, CD90, and CD105 but not CD14, CD19, CD34, or HLA-DR (Fig. S1D). These results are in accordance with the well-established markers of hPMSCs. The hPMSCs displayed typical fibroblastic morphology (Fig. S1A). The hPMSCs were cultured in adipogenic and osteogenic induction medium to differentiate into adipocytes and osteoblasts, respectively. Osteoblasts were verified by Alizarin Red staining of intracellular calcium deposits (Fig. S1B). Oil Red O staining of fat globules was performed in adipocyte induction medium to verify the presence of adipocytes (Fig. S1C).

### Naive CD4^+^ T cell isolation and co-culture with hPMSCs

Human naive CD4^+^ T (CD4CD45RA) cells were prepared using a naive CD4^+^ T cell isolation kit II. CD4^+^ T cells were pretreated for 1 h with the Akt inhibitor LY294002 (30 μM) or Nrf2 inhibitor ML385 (10 μM). In direct co-cultures, hPMSCs were added at a 1:10 ratio to CD4^+^ T cells (4 × 10^6^) in direct contact and cultured at 37 °C in 5% CO_2_ for 72 h in the absence or presence of anti-CD3/CD28 Dynabeads (1 μg/ml) and IL-2 (2.5 ng/ml) as a mitogenic stimulus. In transwell cultures, hPMSCs (4 × 10^5^) were seeded in the upper chamber whereas CD4^+^ T cells (4 × 10^6^) were seeded in the lower chamber.

### Intracellular ROS detection

The intracellular production of ROS was assessed by 2′,7′-dichlorofluorescein diacetate (H2DCF-DA) (Sigma, St. Louis, MO, USA) [19]. CD4^+^ T cells that were not labeled with H2DCF-DA probe used as the negative control group. Briefly, collected CD4^+^ T cells were washed with PBS and then added H2DCF-DA (10 μM) and incubated at 37 °C for 30 min. The levels of intracellular ROS in CD4^+^ T cells were analyzed by FCM after being washed with PBS.

### Antioxidant biomarkers detection

The activities of the antioxidant enzymes superoxide dismutase (SOD), glutathione peroxidase (GSH-Px), and catalase (CAT) in CD4^+^ T cells were measured by colorimetric analysis [20]. GSH-Px activity was detected using the DTNB method [21], CAT activity was measured using the ammonium molybdate method [22], and SOD activity was measured using the xanthine-oxidase method [23].

### SA-β-gal staining

SA-β-Gal activity in senescent T cells was tested as described previously [24]. Briefly, T cells were fixed with 3% formaldehyde ether washed in PBS, and followed to incubate overnight at 37 °C with SA-β-Gal staining solution. After washing with PBS, senescent cells were observed under microscopy (Leica, Germany).

### Western blot analysis

Western blot analyses were performed as previously described [25]. In short, total proteins were electro-transferred to PVDF membranes after being separated by SDS-PAGE. Then, membranes were incubated with indicated primary antibody overnight at 4 °C after being blocked in 5% BSA dissolved in TBST for 2 h at room temperature, followed by incubation with appropriate secondary antibody 2 h at room temperature. ECL plus detection reagents (Beyotime, Shanghai, China) was used for visualized protein bands. The Image J gel analysis software was used for densitometric analysis.

### RNA extraction and quantitative real-time PCR

The levels of mRNA were measured using quantitative real-time PCR assay. Relative abundance of genes was calculated using 2^−∆∆CT^ formula, and β-actin as internal control. Primers attached in the Additional file 1.

### Immunofluorescence assay

CD4^+^ T cells were fixed in 4% paraformaldehyde for 10 min after being washed with cold PBS. Then, cells were permeabilized for 15 min using 1% Triton X-100. After being washed with PBS, cells were incubation with primary antibody overnight. After being washed with PBS, cells were incubation in fluorescence-tagged secondary antibody for 1 h. Nuclei were counter-stained with DAPI. Laser scanning confocal microscope (FV3000, Olympus Corporation, Japan) was used for fluorescence images capture.

### Statistical analysis

Data represent as mean ± SEM. Statistical significance is determined by unpaired two-tailed Student’s *t* test (or nonparametric test) and one-way analysis of variance (ANOVA) followed by Tukey’s post hoc test. Significance was defined as *P* < 0.05.

## Results

### hPMSC treatment attenuates d-gal-induced CD4^+^ T cells senescence in mice

The schematic of hPMSC treatment is shown in Fig. 1a. We purified CD4^+^ T cells using immunomagnetic beads and detected ROS changes in CD4^+^ T cells and the effect of hPMSCs on senescence. The ROS level markedly increased in CD4^+^ T cells collected from d-gal group compared with the control group, while hPMSC treatment significantly decreased the generation of ROS in comparison with that of the PBS treatment group (Fig. 1b–d). In addition, the activities of SOD, CAT, and GSH-Px decreased significantly in the d-gal group compared with the control group, while hPMSC treatment greatly improved the activities of SOD, CAT, and GSH-Px in comparison with that of the PBS treatment group (Fig. 1e–g).

SA-β-gal is considered a key indicator of aging cells [26, 27]. To explore CD4^+^ T cell senescence in an aging mouse model and the effect of hPMSCs on senescence, we detected the positive rate of CD4^+^ T cells using a SA-β-gal kit. The number of SA-β-gal-positive CD4^+^ T cells markedly increased in the d-gal group in comparison with that of the control group. Conversely, the number of SA-β-gal-positive CD4^+^ T cells was markedly declined in hPMSCs group compared with that of PBS treatment group (Fig. 1h, i).

Moreover, the expression of aging-related protein expression of P21 and P16 significantly increased in the d-gal group compared with the control group, while hPMSC treatment greatly declined the levels of P16 and P21 in comparison with that of the PBS treatment group (Fig. 1j, k).

Secretion of proinflammatory factors is thought to be a key feature of SASP phenotype, including IL-8, IL-6, and proinflammatory chemokines [28], and increased expression osteopontin (OPN) was found in senescent CD4^+^ T cells [29]. The mRNA levels of IL-6 and OPN was markedly increased in the d-gal group, while hPMSC treatment greatly declined the levels of IL-6 and OPN in comparison with that of the PBS treatment group (Fig. 1l). These data indicated that hPMSC treatment attenuates CD4^+^ T cell senescence in a d-gal-induced aging mouse model.

### hPMSCs promote the expression of Nrf2-mediated antioxidant genes

**Fig. 2:**
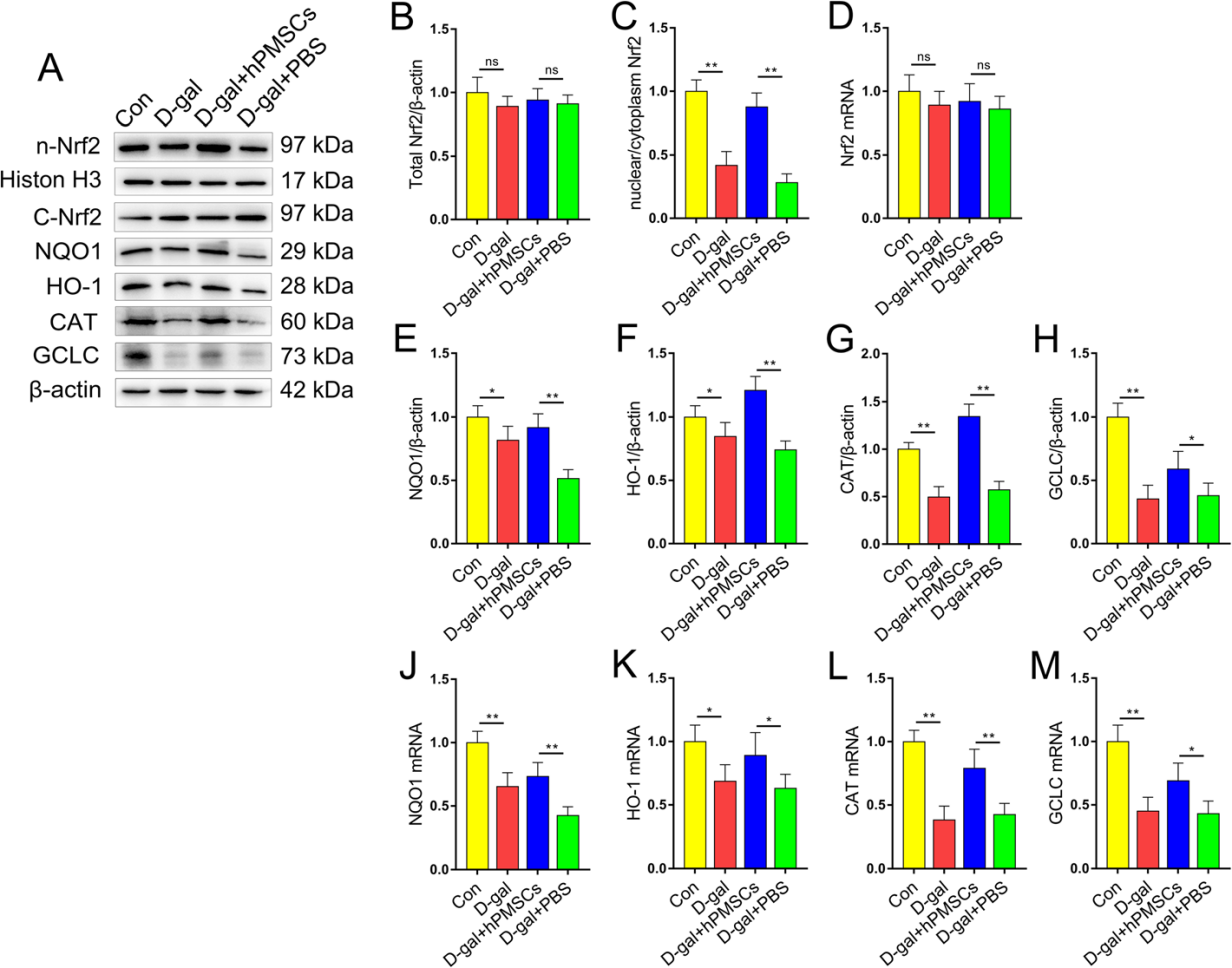
hPMSCs promote the expression of Nrf2-mediated antioxidant genes in CD4^+^ T cells from d-gal-treated mice. **a** The expressions of nucleoprotein and cytosolic protein of Nrf2 and its downstream target genes (GCLC, HO-1, CAT, and NQO1) in CD4^+^ T cells. **b** The protein level of total Nrf2. **c** The ratio of nuclear/cytoplasm Nrf2. **d** The mRNA expressions of Nrf2 in CD4^+^ T cells. **e**–**h** The protein level of Nrf2 downstream target genes GCLC, HO-1, CAT, and NQO1 in CD4^+^ T cells. **j**–**m** The mRNA expressions of GCLC, HO-1, CAT, and NQO1 in CD4^+^ T cells. Data represent the mean scores ± SEM of at least three independent experiments. *p < 0.05, **p < 0.01 (*n* = 10 animals per group)

Nrf2 plays an important role in regulating inflammation, senescence, and intracellular redox balance [30]. Here, we found no significant change in total Nrf2 protein and/or Nrf2 mRNA expression in CD4^+^ T cells under any treatment condition (Fig. 2a, b, d). However, when treated with d-gal, the ratio of nuclear/cytoplasm Nrf2 was markedly declined in CD4^+^ T cells (Fig. 2a, c). Conversely, hPMSC treatment markedly increased the ratio of nuclear/cytoplasm Nrf2 in CD4^+^ T cells compared with those of the PBS treatment group (Fig. 2a, c). These results suggested that hPMSC treatment upregulates the nuclear transfer of Nrf2 rather than increase the expression of total Nrf2. In addition, we found that the protein and/or mRNA expression of the Nrf2 target antioxidant genes NQO1, CAT, HO-1, and GCLC was markedly lower in the d-gal group compared with the control group (Fig. 2a, e–h). Conversely, hPMSC treatment greatly increased the expression of these proteins and/or mRNAs in comparison with that of the PBS treatment group (Fig. 2a, e–h). These data indicated that hPMSC treatment promoted the nuclear transfer of Nrf2 and the expression of Nrf2 target antioxidant genes.

### hPMSCs upregulate the expression of Nrf2 via the Akt/GSK-3β/Fyn pathway

**Fig. 3:**
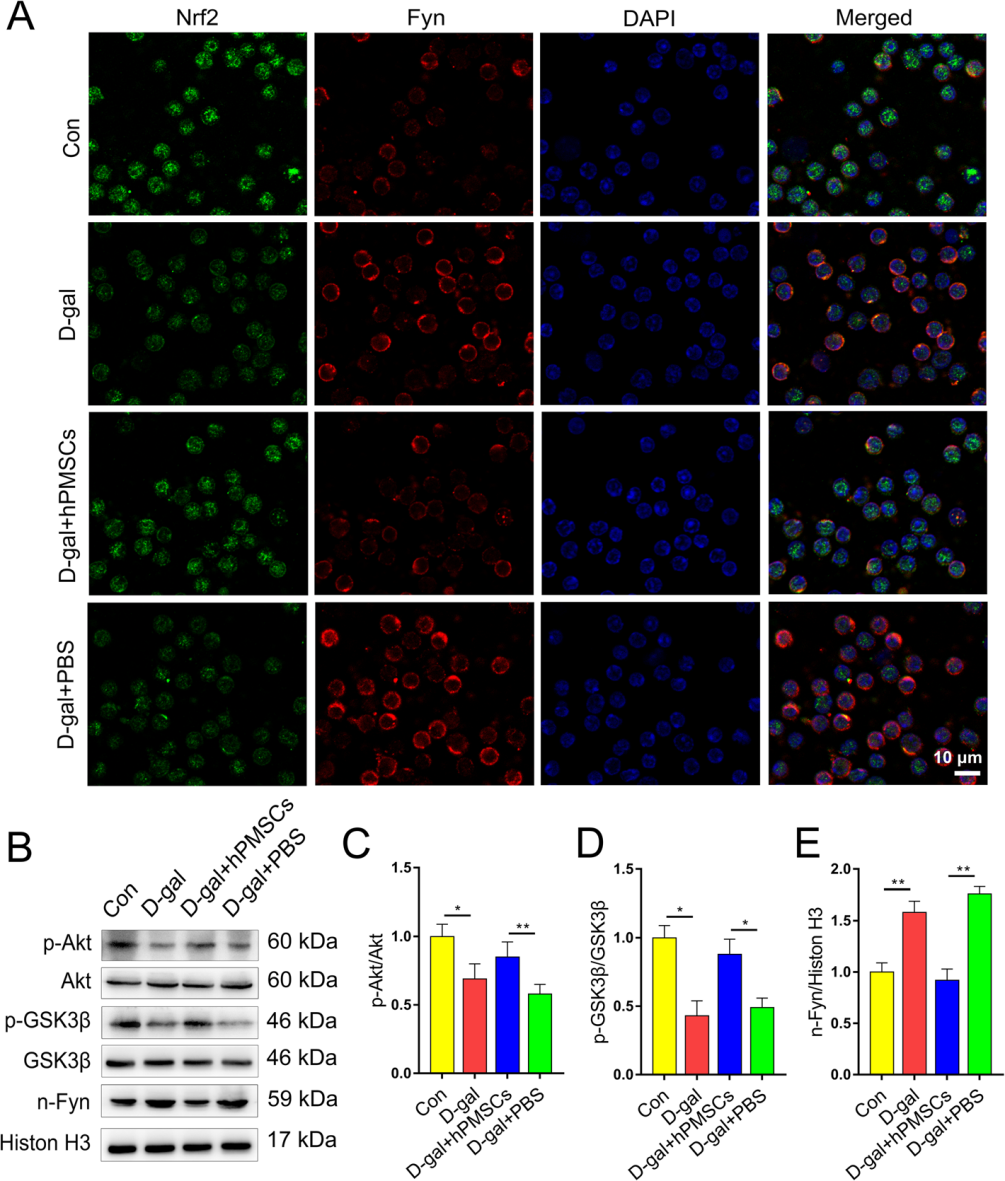
hPMSCs upregulate the expression of Nrf2 via Akt/GSK-3β/Fyn pathway in CD4^+^ T cells from d-gal-treated mice. **a** Nuclear translocation of Nrf2 and Fyn were determined by immunofluorescent staining (bar = 10 μm). **b** The expressions of Akt/GSK-3β/Fyn pathway in CD4^+^ T cells. **c** The ratio of p-Akt/Akt. **d** The ratio of p-GSK-3β/GSK-3β. **e** The level of nuclear Fyn. Data represent the mean scores ± SEM of at least three independent experiments. *p < 0.05, **p < 0.01 (*n* = 10 animals per group)

Previous studies indicated that the activation of Nrf2 is regulated by adjusting Fyn-mediated degradation and nuclear export of Nrf2 [31]. To investigate the mechanisms by which hPMSC treatment activates Nrf2 transcriptional functions in CD4^+^ T cells, the total and phosphorylated GSK-3β, Akt, and nuclear Fyn levels was measured. As shown in Fig. 3b–e, we found markedly decreased phosphorylation of GSK-3β and Akt and increased nucleus Fyn level in the d-gal group. hPMSC treatment markedly raised the phosphorylation of GSK-3β and Akt and decreased the level of Fyn in the nucleus in comparison with those of the PBS group. These results were also supported by examining Nrf2 and Fyn nuclear localization in the different groups (Fig. 3a). These results suggest that hPMSCs attenuate CD4^+^ T cell senescence by upregulating Nrf2 functions via Akt/GSK-3β/Fyn pathway.

### hPMSCs alleviates CD4^+^ T cell senescence in vitro

**Fig. 4:**
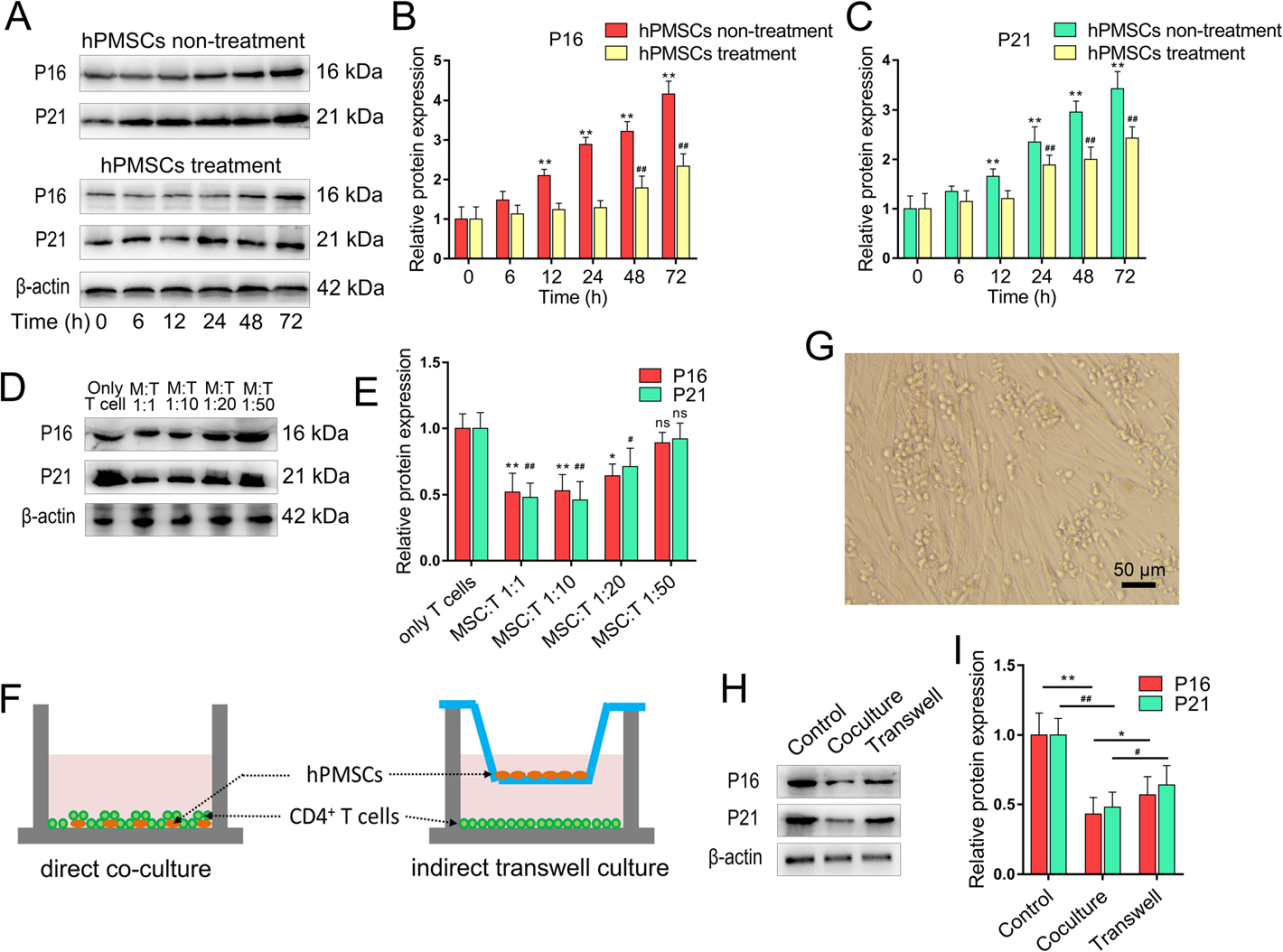
hPMSCs alleviates CD4^+^ T cell senescence in vitro*.* 4 × 10^6^ human naive CD4^+^ T cells (CD4CD45RA cells) were isolated and co-cultured with hPMSCs (4 × 10^5^) for 72 h in the presence of anti-CD3/CD28 Dynabeads and IL-2 as a mitogenic stimulus. **a**–**c** The expressions of senescent markers P16 and P21 in CD4^+^ T cells under hPMSC treatment or non-treatment condition. **d**, **e** The expression of P16 and P21 in CD4^+^ T cells were detected after being co-cultured with hPMSCs at different ratios (T cells only, hPMSCs: T cells = 1:1, 1:10, 1:20, and 1:50). **f** A schematic of the experiments. In transwell cultures, hPMSCs (4 × 10^5^) were seeded on the upper chamber and CD4^+^ T cells (4 × 10^6^) were in the lower chamber. **g** An image of the direct co-culture of hPMSCs with CD4^+^ T cells under a light microscope. **h**, **i** The expressions of P16 and P21 in CD4^+^ T cells in different co-culture conditions. Data represent the mean scores ± SEM of at least three independent experiments. *p < 0.05, **p < 0.01

We next examined the protective effect of hPMSCs on the aging process of CD4^+^ T in vitro. A total of 4 × 10^6^ human naive CD4^+^ T cells (CD4CD45RA cells) were isolated and co-cultured with hPMSCs (4 × 10^5^) for 72 h in the presence of anti-CD3/CD28 Dynabeads and IL-2 as a mitogenic stimulus. As shown in Fig. 4a and b, with the increase of culture time, the expression of P16 and P21 raised in activated naive CD4^+^ T cells. However, the increase rate of P16 and P21 expression in activated naive CD4^+^ T cells was significantly reduced after hPMSC treatment. And the difference of the P16 and P21 expression was increased between hPMSC treatment and non-treatment groups with a time-dependent manner. In addition, the expression of P16 and P21 in CD4^+^ T cells were also detected after being co-cultured with hPMSCs at different ratios (T cells only, hPMSCs: T cells = 1:1, 1:10, 1:20, and 1:50) to explore the dose-dependent effect of hPMSC treatment. As shown in Fig. 4d and e, the effect of hPMSCs in alleviating CD4^+^ T cells senescence showed dose-dependence, and the therapeutic effect was gradually weakened when the cells ratio was more than 1:10. Furthermore, to explore the anti-aging effect of hPMSCs on CD4^+^ T cells was cell-cell contact dependent or not. Here, we tested the expression of P16 and P21 in CD4^+^ T cells after indirect (Transwell) or direct co-culture with hPMSCs for 72 h (Fig. 4f, g). As shown in Fig. 4h and i, we found that hPMSCs attenuate CD4^+^ T cells senescence under both culture conditions. In addition, the protective effect of contact co-culture was better than that of indirect transwell co-culture.

### Inhibition of Akt/GSK-3β/Fyn pathway downregulates the expression of Nrf2-regulated antioxidant genes in senescent CD4^+^ T cells

**Fig. 5:**
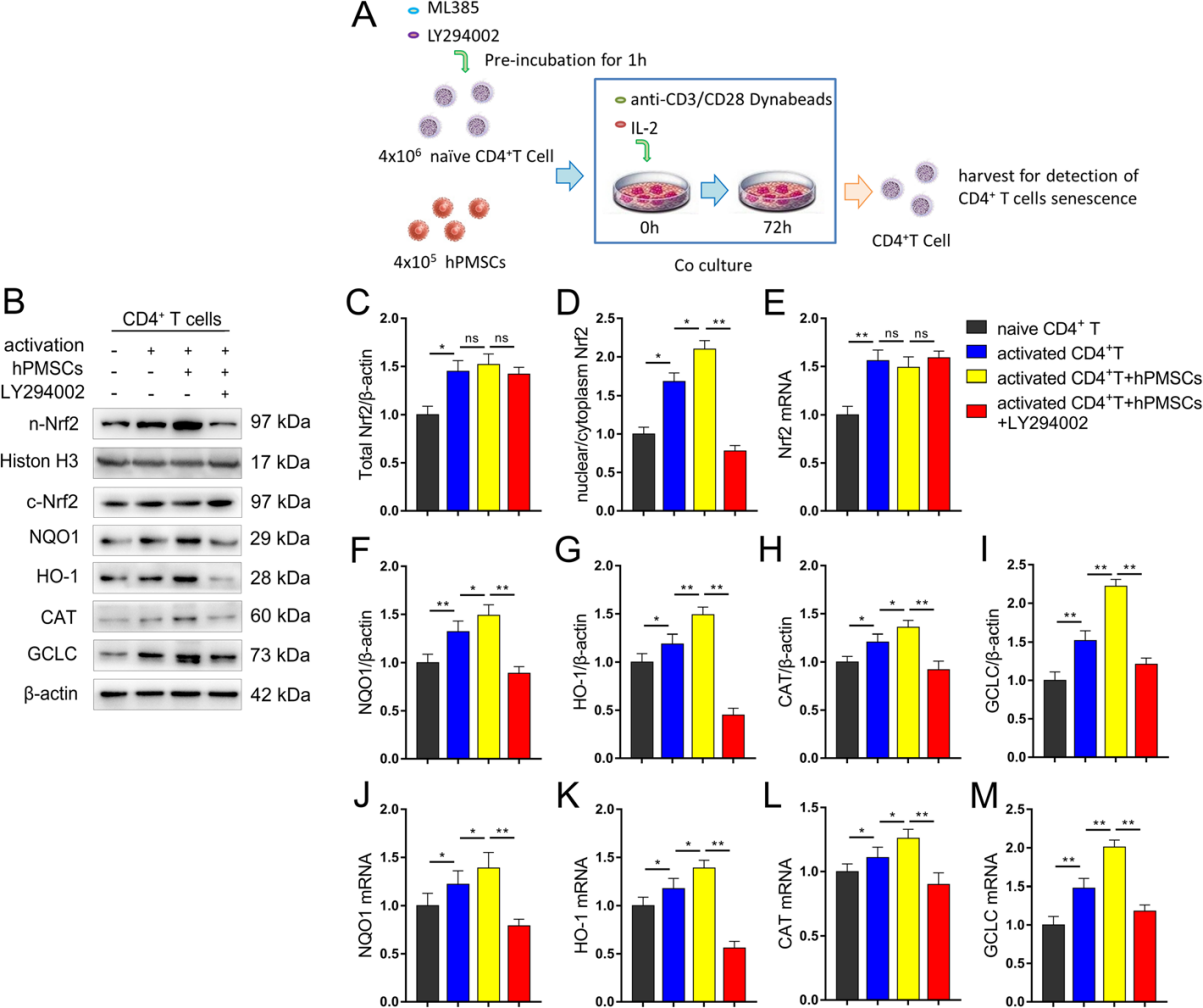
hPMSCs upregulate the expression of Nrf2 of senescent CD4^+^ T cells. 4 × 10^6^ naive CD4^+^ T cells (CD4CD45RA cells) were isolated using immunomagnetic beads and pretreatment for 1 h with Akt pathway inhibitor LY294002 (30 μM). Then, hPMSCs were added at a 1:10 ratio to CD4^+^ T cells and co-cultured for 72 h in the presence or absence of anti-CD3/CD28 Dynabeads and IL-2 as a mitogenic stimulus. **a** The scheme showing the experimental design. **b** Protein levels of Nrf2 and its downstream target genes NQO1, HO-1, CAT, and GCLC. **c** The level of total Nrf2. **d** The ratio of nuclear/cytoplasm Nrf2. **e** The mRNA expressions of Nrf2 in CD4^+^ T cells. **f**–**i** The protein level of Nrf2 downstream target genes GCLC, HO-1, CAT, and NQO1 in CD4^+^ T cells. **j**–**m** The mRNA expressions of GCLC, HO-1, CAT, and NQO1 in CD4^+^ T cells. Data represent the mean scores ± SEM of at least three independent experiments. *p < 0.05, **p < 0.01

**Fig. 6:**
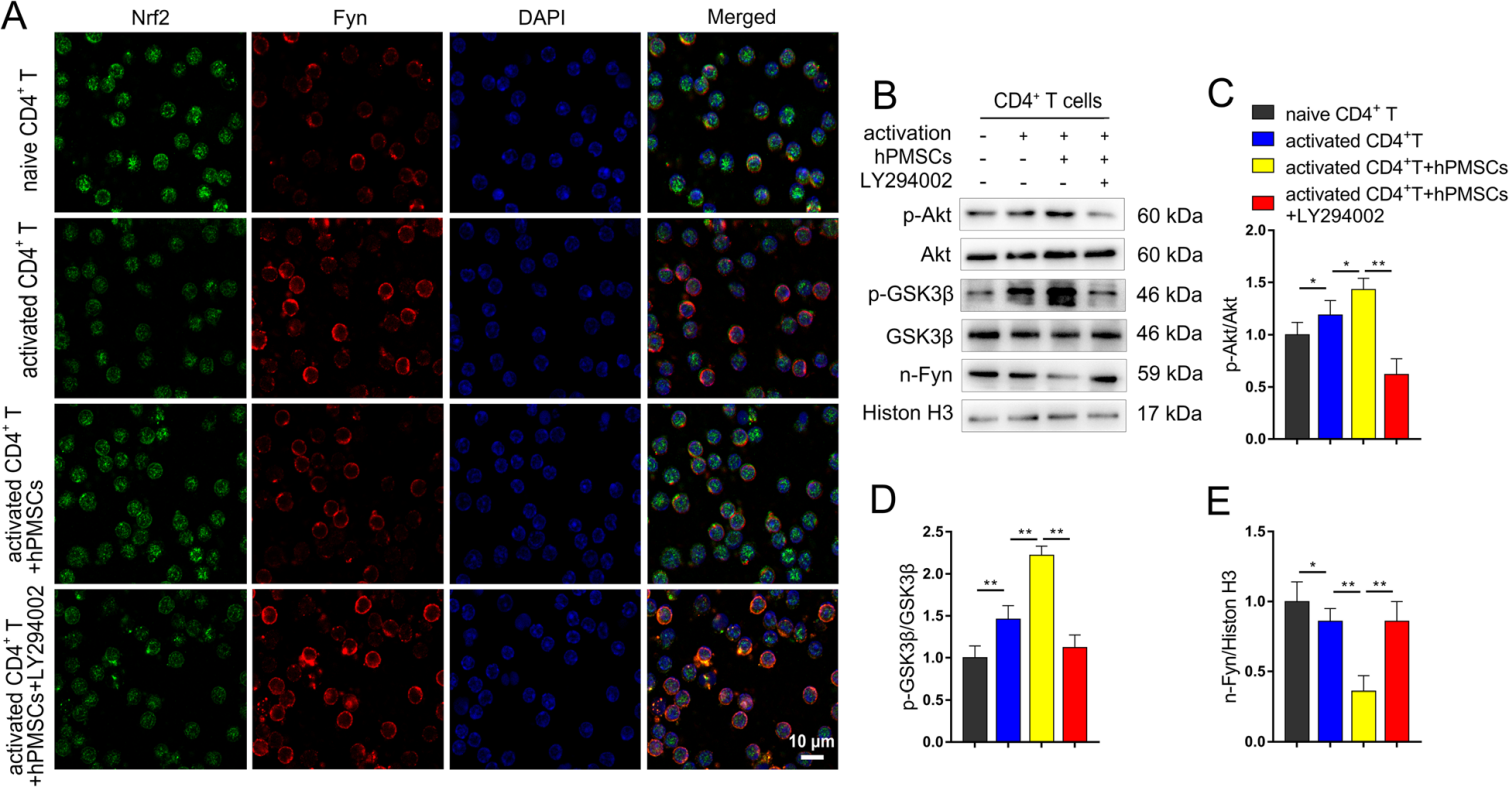
Inhibition of Akt/GSK-3β/Fyn pathway downregulate the expression of Nrf2-regulated antioxidant genes in senescent CD4^+^ T cells. **a** Nuclear translocation of Nrf2 and Fyn were determined by immunofluorescent staining (bar = 10 μm). **b** The expressions of Akt/GSK-3β/Fyn pathway in CD4^+^ T cells were evaluated by Western blot. **c** The ratio of p-Akt/Akt. **d** The ratio of p-GSK-3β/GSK-3β. **e** The level of nuclear Fyn. Data represent the mean scores ± SEM of at least three independent experiments. *p < 0.05, **p < 0.01

We further confirmed whether the Akt/GSK-3β/Fyn pathway was critical in the protective effects observed in senescent CD4^+^ T cells that were treated with hPMSCs. A total of 4 × 10^6^ human naive CD4^+^ T cells (CD4CD45RA cells) were isolated and pretreated for 1 h with the Akt inhibitor LY294002 (30 μM). Then, hPMSCs were added at a 1:10 ratio to CD4^+^ T cells and co-cultured for 72 h in the presence or absence of anti-CD3/CD28 Dynabeads and IL-2 as a mitogenic stimulus. The schematic showing the experimental design is shown in Fig. 5a. As shown in Fig. 5b–d, the ratio of nuclear/cytoplasm Nrf2 markedly improved in activated CD4^+^ T cells after being cultured for 72 h, while hPMSC treatment further improved the ratio of nuclear/cytoplasm Nrf2 in activated CD4^+^ T cells. Furthermore, we found that the mRNA and/or protein expression of the Nrf2 target antioxidant genes NQO1, HO-1, CAT, and GCLC were also markedly improved in activated CD4^+^ T cells (Fig. 5f–m). hPMSC treatment further increased the expression of these proteins and/or mRNAs in activated CD4^+^ T cells (Fig. 5f–m). Moreover, we found markedly increased phosphorylation of GSK-3β and Akt and decreased nucleus Fyn level in activated CD4^+^ T cells (Fig. 6b–e). hPMSC treatment significantly increased the phosphorylation of GSK-3β and Akt and decreased the nuclear Fyn level (Fig. 6b–e). These results were also supported by examining Nrf2 and Fyn nuclear localization in the different groups (Fig. 6a).

Subsequently, we explored the impact of LY294002 on the expression of Nrf2 and its downstream target genes in the presence of hPMSCs. Significantly reduced nuclear Nrf2, NQO1, HO-1, CAT, and GCLC expression was observed in hPMSC treated CD4^+^ T cells after LY294002 supplementation (Fig. 5b–m). These data revealed that the effects of hPMSCs on Nrf2-mediated antioxidant signal are downstream of the Akt/GSK-3β/Fyn pathway in senescent CD4^+^ T cells.

### Inhibition of the Akt/GSK-3β/Fyn pathway impairs the protective effects of hPMSCs on senescent CD4^+^ T cells

**Fig. 7:**
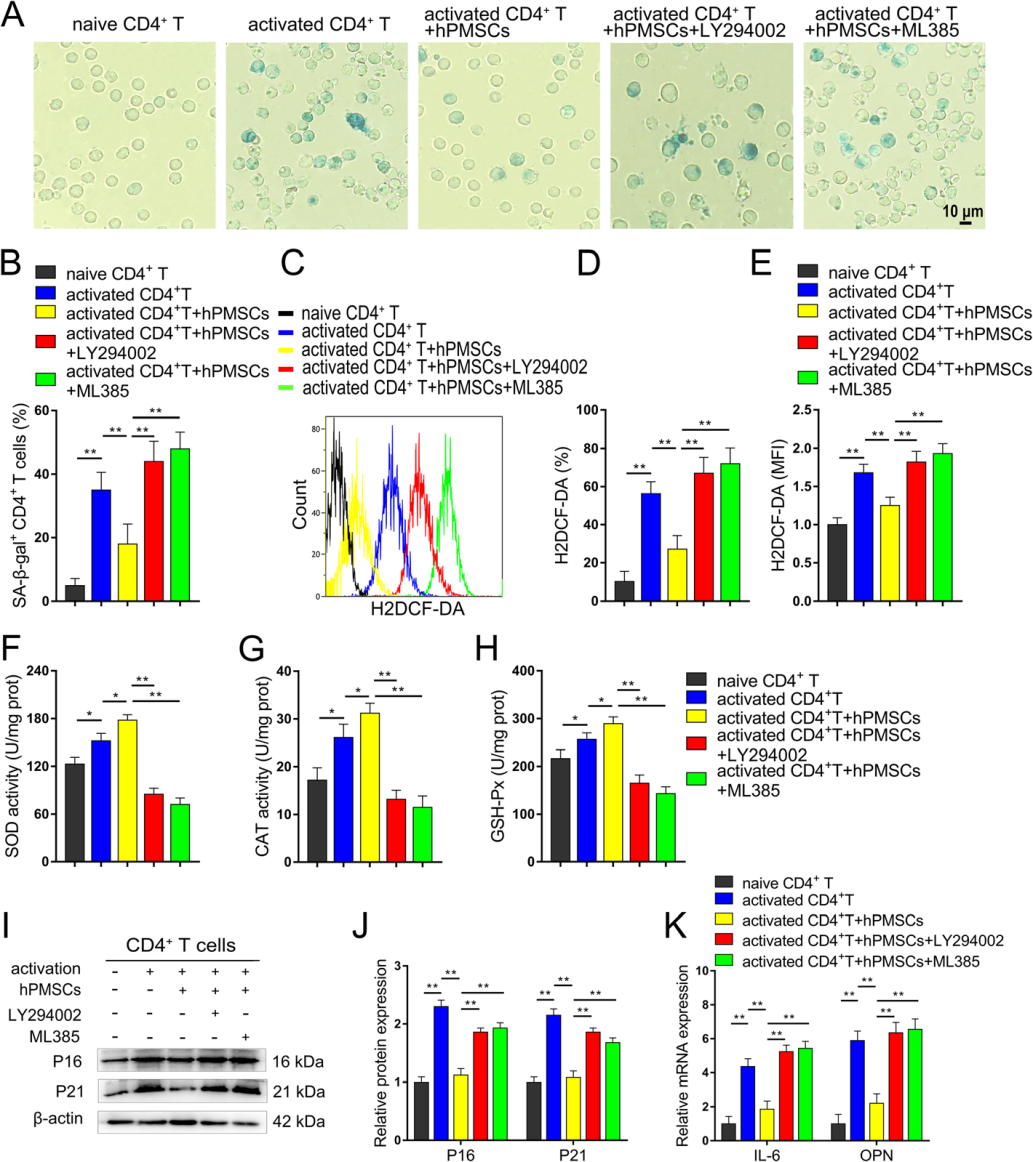
Inhibition of Akt/GSK-3β/Fyn pathway impairs the protective effects of hPMSCs in senescent CD4^+^ T cells. **a**, **b** The percentages of SA-β-gal-positive CD4^+^ T cells are shown (bar = 10 μm). **c**, **e** The expression of ROS in CD4^+^ T cells. **f**–**h** The antioxidant enzyme activity of SOD, CAT, and GSH-Px in CD4^+^ T cells. **i**, **j** The expressions of senescent markers P16 and P21 in CD4^+^ T cells. **k** The mRNA expressions of IL-6 and OPN in CD4^+^ T cells. Data represent the mean scores ± SEM of at least three independent experiments. *p < 0.05, **p < 0.01

To further elucidate the protective effects of hPMSCs on senescent CD4^+^ T cells by Akt-mediated Nrf2 antioxidant signaling, we conducted SA-β-gal staining and detected changes in ROS in CD4^+^ T cells after co-culture with hPMSCs. As shown in Fig. 7a–e, hPMSC treatment significantly decreased the percentage of SA-β-gal-positive CD4^+^ T cells and the level of ROS in activated CD4^+^ T cells. Although the antioxidant enzyme activity of SOD, CAT, and GSH-Px were improved significantly in CD4^+^ T cells after being activated, hPMSC treatment could further improve the antioxidant enzyme activity in activated CD4^+^ T cells (Fig. 7f–h). Furthermore, the significantly decreased percentage of SA-β-gal-positive CD4^+^ T cells and ROS levels and significantly increased antioxidant enzyme activity were abolished by LY294002 or ML385 (Nrf2 inhibitor) supplementation. Moreover, the hPMSC-mediated reduction in the expression of aging-related mRNA and/or protein for IL-6, OPN, P16, and P21 were also abrogated by LY294002 or ML385 supplementation (Fig. 7i–k). These data revealed that the protective effects of hPMSCs on senescent CD4^+^ T cells were dependent on Akt-mediated Nrf2 antioxidant signaling.

## Discussion

CD4^+^ T cells play a central role in the persistence and development of immune responses. Evidence indicates that increased oxidative damage is related to the aging-related decline in immune functions [4, 32]. Based on the antioxidant and anti-aging effects stem cells have been thought to be the source of seed cells for tissue engineering and biological therapeutics [16, 33]. However, there is no evidence showing the protective effect of MSCs against aging-induced T cell dysfunction. Therefore, the present study shows that (1) hPMSCs attenuate CD4^+^ T cell senescence with a time and dose-dependent manner, (2) hPMSCs played a protective effect by upregulating Nrf2-mediated antioxidant signal in senescent CD4^+^ T cells, and (3) hPMSC treatment activate the Akt/GSK-3β/Fyn pathway resulting in inhibition of Fyn-mediated degradation of Nrf2, which improved the nuclear translocation of Nrf2 and the expression of downstream target antioxidant genes (GCLC, HO-1, CAT, and NQO1) in senescent CD4^+^ T cells.

The balance between ROS generation and antioxidant capacity is necessary to ensures physiological levels of intracellular ROS and T cell-mediated immune response. Accordingly, excessive ROS generation in T cells will destroy the intracellular redox balance and leads to metabolism disorder and immune response dysfunction [19, 34]. Here, we also observed markedly raised ROS level and decreased antioxidant enzyme activity of SOD, CAT, and GSH-Px in the d-gal-treated group. Senescent cells cause serious damage to the tissue microenvironment by secreting senescence-associated secretory phenotype, which is characterized by a markedly upregulating in the secretion of proinflammatory cytokines, matrix remodeling factors, chemokines, and proangiogenic factors [35, 36]. In this study, we also found markedly increased SASP expression (IL-6 and OPN) in senescent CD4^+^ T cells. Furthermore, the levels of P16 and P21 and SA-β-gal-positive cells increased markedly in senescent CD4^+^ T cells. Our data are consistent with previous findings of the gradual accumulation of P16 and P21 expression and increased activity of SA-β-gal during several aging-associated diseases and physiological aging [37, 38]. Based on the antioxidant capacity, MSCs and the MSC secretome derived from distinct tissue origins have been tested for the treatment of many diseases. Yan et al. found that hfPMSCs protected against H_2_O_2_-induced cell oxidative damage and apoptosis by upregulating Nrf2/Keap1/ARE antioxidant signaling [39]. Shalaby et al. reported that MSC injection was effective in modulating oxidative stress in *E. coli*-induced acute lung injury [40]. Here, we reported that hPMSC treatment markedly decreased the levels of ROS, SASP (IL-6 and OPN), aging-related protein (P16 and P21), and the number of SA-β-gal-positive cells in senescent CD4^+^ T cells. These results indicated that hPMSCs attenuate age-associated CD4^+^ T cell senescence. As a critical redox sensor, Nrf2 plays a vital role in antioxidant response in most tissue cells [41]. Numerous studies have suggested that disrupted activation of Nrf2 antioxidant signaling leading to decreased endogenous antioxidant response during aging [37, 42, 43]. In this study, we found that aging accompanies markedly decreased nuclear accumulation of Nrf2 and decreased expression of downstream antioxidant target genes in CD4^+^ T cells. This may be an important reason for the markedly decreased antioxidant enzyme activity and increased level of intracellular ROS in CD4^+^ T cells of the aging group. Recent studies have found that MSCs exert antioxidant effects by upregulating the Nrf2 pathway. Recently report by Zhang et al. shown that MSCs alleviate inflammatory responses and acute lung injury that are induced by paraquat poisoning by upregulating Nrf2 and activating the downstream antioxidant HO-1 [16]. Ni et al. reported that bone marrow mesenchymal stem cells (BMSCs) attenuated bleomycin-induced oxidative damage via the activation of HO-1, γ-GCS, and NQO1 expression and the Nrf2 pathway [44]. Here, we found that hPMSC treatment effectively upregulated Nrf2 nuclear translocation and the expression of downstream target genes (GCLC, HO-1, CAT, and NQO1) in CD4^+^ T cells. And the antioxidant enzyme activity of SOD, CAT, and GSH-Px were also improved markedly in senescent CD4^+^ T cells after hPMSC treatment. Our results suggested the crucial protective effect of hPMSCs in alleviating ROS-induced CD4^+^ T cells dysfunction by activating Nrf2 antioxidant signaling during aging.

The mechanism of Nrf2 regulation has been widely studied [45, 46]. Several recent studies have suggested that the activation of Nrf2 was regulated by Akt/GSK-3β/Fyn-mediated degradation and nuclear export of Nrf2 [31, 47]. In this study, we detected significantly decreased activation of the Akt/GSK-3β/Fyn pathway in senescent CD4^+^ T cells. Similarly, a d-gal-induced decline in Akt phosphorylation was also found in human umbilical vein endothelial cells [48]. Recently, Li et al. reported that human amniotic MSCs efficiently ameliorate heat stress-induced skin injury by inhibiting apoptosis in skin cells through activating the Akt signaling pathway [49]. Based on these studies, we hypothesized that Akt/GSK-3β/Fyn pathway is involved in hPMSC-induced Nrf2 activation in senescent CD4^+^ T cells. Consistent with this hypothesis, we found that hPMSC treatment not only increased Akt phosphorylation but also inhibited GSK-3β activity, which decreased Fyn nuclear accumulation. Inactivation of Fyn kinase reinforces cell antioxidant defense by abolishes ubiquitination-mediated Nrf2 suppression. Under in vitro, we found that inhibition of the PI3K/Akt pathway by the inhibitor LY294002 downregulated Nrf2-regulated antioxidant genes in senescent CD4^+^ T cells. Moreover, the hPMSC-mediated reduction in the expression of aging-related mRNA and/or protein for IL-6, OPN, P16, and P21 were abolished by Akt inhibitor LY294002 and Nrf2 inhibitor ML385. In addition, the decreased percentage of SA-β-gal-positive CD4^+^ T cells and ROS levels by hPMSC treatment were also abolished by LY294002 and ML385 supplementation. Our findings reveal that the protective effects of hPMSCs on senescent CD4^+^ T cells depend on Akt-mediated Nrf2 antioxidant signaling. However, the direct link between hPMSC treatment and phosphorylation of the Akt pathway in senescent CD4^+^ T cells remains a key unanswered question.

It has been found that direct cell-cell contact between MSCs and CD4^+^ T cells was required for the immunomodulatory effect of MSCs [50, 51]; therefore, we investigated whether cell-cell contact is necessary in order for hPMSCs to display their anti-aging effect on CD4^+^ T cells, by using both indirect (Transwell) and direct co-culture strategies. We found that also hPMSCs attenuate CD4^+^ T cells senescence under both co-culture conditions, it was much weaker in indirect contact than when cells were allowed to have direct contact. This finding fits with the evidence that both cell-cell contact and secreted molecules are necessary for immunomodulatory effect of MSCs on T cells [52]. The unknown molecular regulator(s) might be associated with the contact-dependent mechanism underlying this event should be further studied.

## Conclusions

**Fig. 8:**
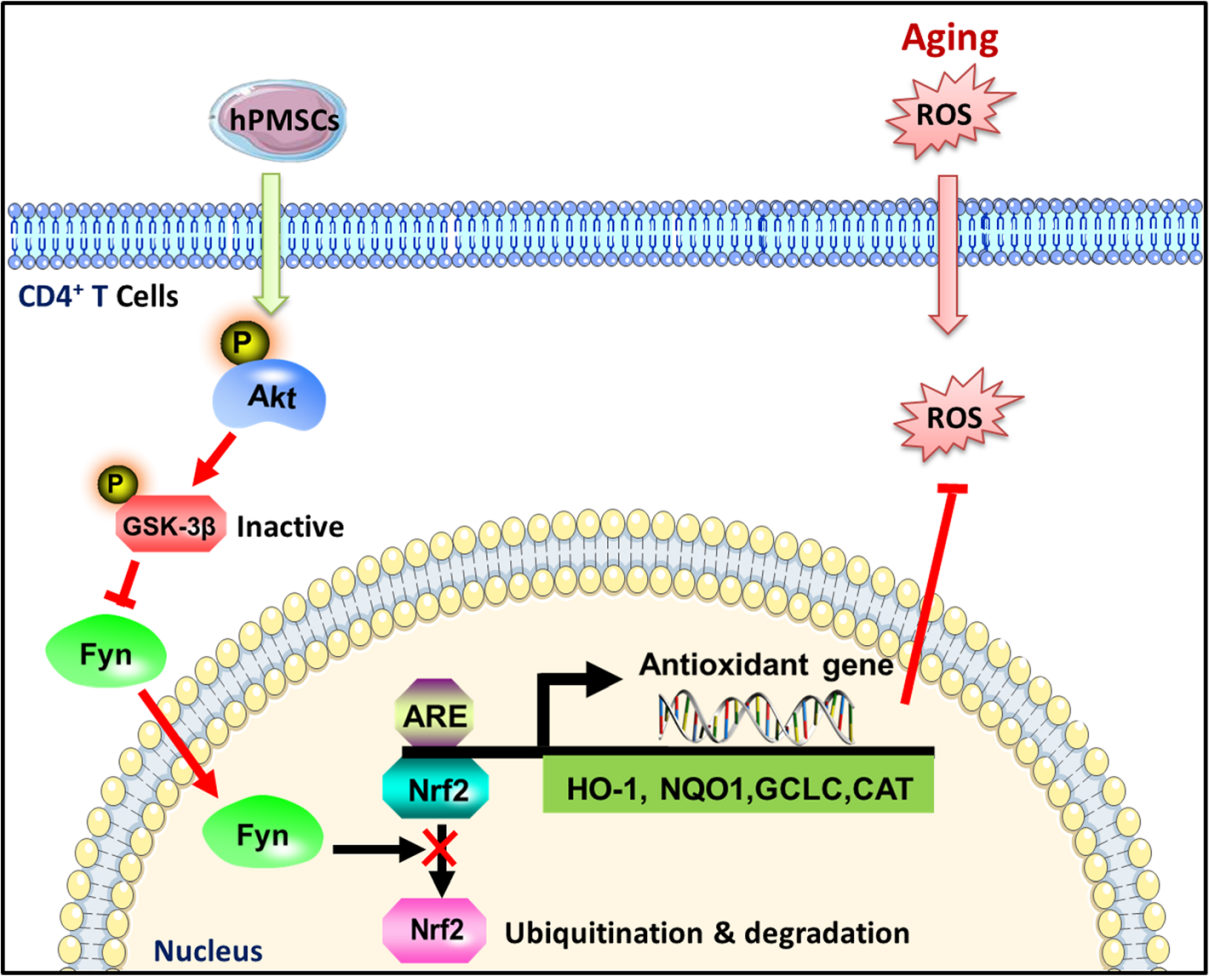
Schematic illustration of the protective effects of hPMSCs on CD4^+^ T cells senescence. Aging is accompanied by decreased activation of Nrf2 antioxidant pathway and dysfunction of redox metabolism in CD4^+^ T cells. hPMSCs attenuate CD4^+^ T cells senescence via activating Nrf2-mediated exogenous antioxidant defenses. hPMSCs improve Nrf2 activation mediated by increasing phosphorylation of Akt pathway and inhibiting Fyn-mediated degradation of Nrf2

In summary, as illustrated in Fig. 8, our data demonstrate a novel role for hPMSCs in attenuating d-gal induced CD4^+^ T cell senescence by activating Nrf2-mediated antioxidant defenses and that upregulation of Nrf2 by hPMSCs is regulated partially via the Akt/GSK-3β/Fyn pathway. Our findings reveal that the administration of hPMSCs may be a novel therapeutic strategy for immunosenescence treatment.

## Supplementary Information

**Additional file 1.** Sequences used for RT-qPCR.

**Additional file 2: Fig. S1.** Immunophenotyping and differentiation of hPMSCs. (A) The hPMSCs showed typical fibroblastic morphology. (B) Osteogenesis of differentiated hPMSCs was confirmed by Alizarin Red staining. (C) Adipogenic differentiated cells were demonstrated by the accumulation of oil droplets that were positively stained for Oil Red O staining (Bar =100 μm). (D) Cell surface markers of hPMSCs analyzed by FCM. The green histograms represented the isotype control. The specific expression of the indicated cell surface markers was presented as red histograms.

## Data Availability

All data generated or analyzed during this study are included in the published article.

## Abbreviations

MSCs: Mesenchymal stem cells
Nrf2: Nuclear factor E2-related factor 2
HO-1: Heme oxygenase-1
NQO1: NAD(P) H quinone oxidoreductase 1
GCLC: Catalytic subunits of glutamate cysteine ligase
Akt: Protein kinase B
GSK-3β: Glycogen synthase kinase 3β
SASP: Senescence-associated secretory phenotype
ROS: Reactive oxygen species
OPN: Osteopontin
IL-6: Interleukin-6
d-gal: d-galactose
CAT: Catalase
