# Correction: hPMSCs protects against D-galactose-induced oxidative damage of CD4^+^ T cells through activating Akt-mediated Nrf2 antioxidant signaling

**DOI:** 10.1186/s13287-024-04045-z

**Published:** 2024-11-07

**Authors:** Yanlian Xiong, Yueming Wang, Jiashen Zhang, Nannan Zhao, Hengchao Zhang, Aiping Zhang, Dongmei Zhao, Zhenhai Yu, Yancun Yin, Lele Song, Yanlei Xiong, Xiying Luan

**Affiliations:** 1 Department of Anatomy, School of Basic Medicine, Binzhou Medical University, Yantai, People’s Republic of China; 2 Department of Immunology, School of Basic Medicine, Binzhou Medical University, Yantai, People’s Republic of China; 3 Department of Pathology, Xuanwu Hospital, Capital Medical University, Beijing, People’s Republic of China

**Correction to: Stem Cell Research & Therapy (2020) 11:468**

10.1186/s13287-020-01993-0

Following the publication of the original article, the authors identified an error in the article.

The authors noticed that the gel bands of β-actin (Fig. 1J) and p-GSK3β (Fig. 6B) were wrongly used during the process of image analysis and comparison.

It has been corrected after they double-checked the original data. These corrections will not affect the result and conclusion of the article.

The authors sincerely apologize for any inconvenience caused.

**Fig. 1:**
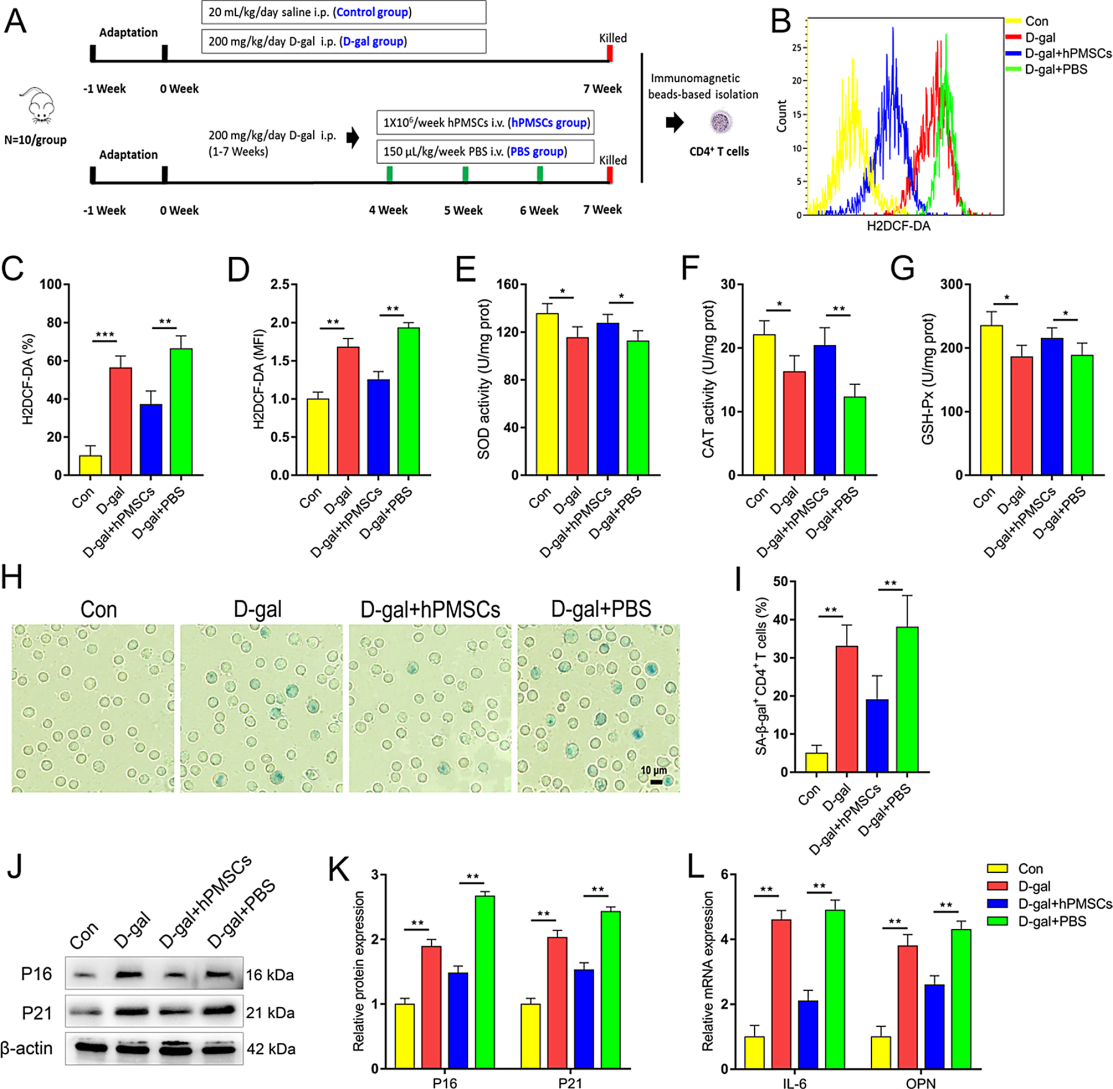
hPMSCs relieves D-gal triggered CD4^+^ T cells senescence in mice. (**A**) The scheme of the experimental design. (**B**-**D**) The expression of ROS in CD4^+^ T cells. (**E**-**G**) The antioxidant enzyme activity of SOD, CAT and GSH-Px in CD4^+^ T cells. (**H**-**I**) The percentages of SA-β-gal-positive CD4^+^ T cells are shown (Bar = 10 μm). (**J**-**K**) The expressions of senescent markers P16 and P21 in CD4^+^ T cells. (**L**) The mRNA expressions of IL-6 and OPN in CD4^+^ T cells. Data represent the mean scores ± SEM of at least three independent experiments. *p<0.05, **p<0.01, (*n* = 10 animals per group)

**Fig. 6:**
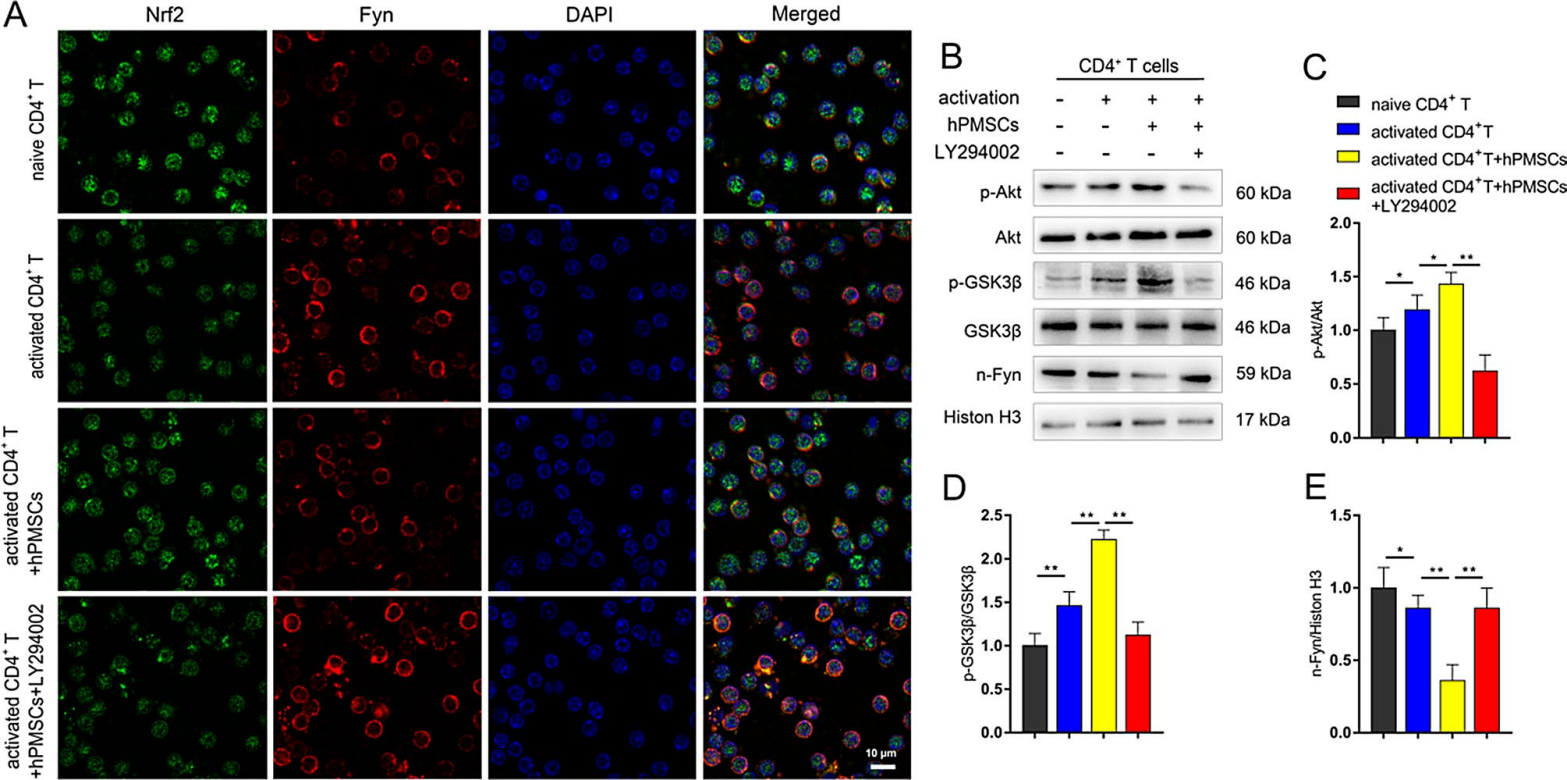
Inhibition of Akt/GSK-3β/Fyn pathway downregulate the expression of Nrf2-regulated antioxidant genes in senescent CD4^+^ T cells. (**A**) Nuclear translocation of Nrf2 and Fyn were determined by immunofluorescent staining (Bar = 10 μm). (**B**) The expressions of Akt/GSK-3β/Fyn pathway in CD4^+^ T cells were evaluated by Western blot. (**C**) The ratio of p-Akt/Akt. (**D**) The ratio of p-GSK-3β/GSK-3β. (**E**) The level of nuclear Fyn. Data represent the mean scores ± SEM of at least three independent experiments. *p<0.05, **p<0.01

