# Supplementary material for: hPMSCs protects against d-galactose-induced oxidative damage of CD4+ T cells through activating Akt-mediated Nrf2 antioxidant signaling
Source: Stem Cell Res Ther. 2020 Nov 4;11:468. doi: 10.1186/s13287-020-01993-0 (PMC7641865; doi:10.1186/s13287-020-01993-0)
Supplement: Supplementary file 1 — Additional file 1. Sequences used for RT-qPCR. [file 13287_2020_1993_MOESM1_ESM.docx]

| Name | Primer Sequences (Forward/Reverse Primer) |
| --- | --- |
| Nrf2 | CAGCATAGAGCAGGACAT  GGAACAGCGGTAGTATCA |
| NQO1 | TTTGAGAGAGTGCTCGTAGC  GGTCTTCTTATTCTGGAAAGG |
| HO-1 | CCCAGTCTATGCCCCACTCT  AGACGCTTTACATAGTGCTG |
| GCLC | AACAAGAAACATCCGGCATC  CGTAGCCTCGGTAAAATGGA |
| CAT | CCACAGTCGCTGGAGAGTCA  GTTTCCCACAAGGTCCCAGTT |
| IL-6 | ACATCCTCGACGGCATCTCA  TCACCAGGCAAGTCTCCTCA |
| OPN | TTGCAGCCTTCTCAGCCAA  CAAAAGCAAATCACTGCAATTCTC |
| β-actin | CACCACACCTTCTACAATGAG  TACGACCAGAGGCATACAG |

Additional file 1. Sequences used for RT-qPCR
