# Supplementary figures and images for: hPMSCs protects against d-galactose-induced oxidative damage of CD4+ T cells through activating Akt-mediated Nrf2 antioxidant signaling
Source: Stem Cell Res Ther. 2020 Nov 4;11:468. doi: 10.1186/s13287-020-01993-0 (PMC7641865; doi:10.1186/s13287-020-01993-0)

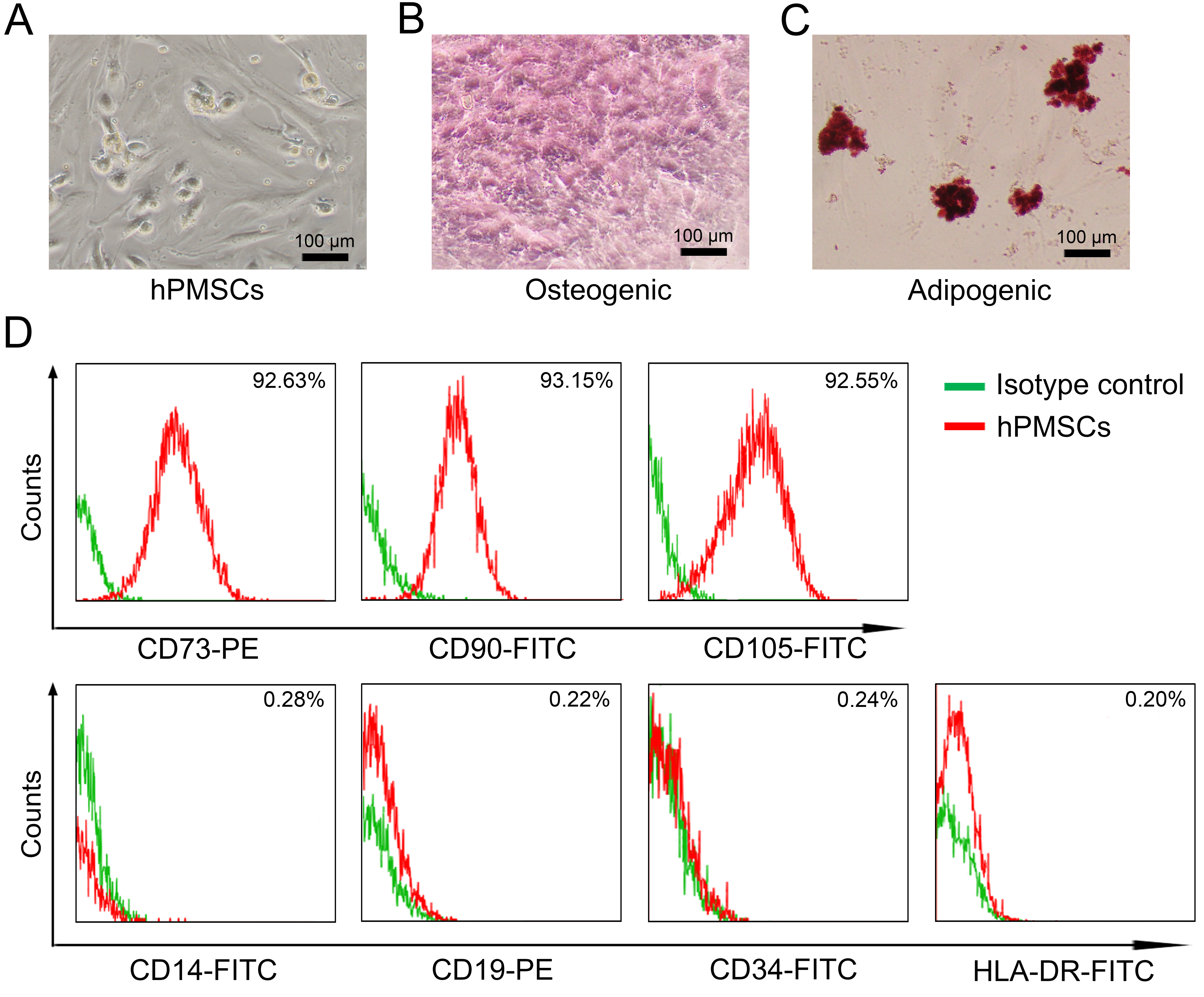

Supplement: Supplementary file 2 — Additional file 2: Fig. S1. Immunophenotyping and differentiation of hPMSCs. (A) The hPMSCs showed typical fibroblastic morphology. (B) Osteogenesis of differentiated hPMSCs was confirmed by Alizarin Red staining. (C) Adipogenic differentiated cells were demonstrated by the accumulation of oil droplets that were positively stained for Oil Red O staining (Bar =100 μm). (D) Cell surface markers of hPMSCs analyzed by FCM. The green histograms represented the isotype control. The specific expression of the indicated cell surface markers was presented as red histograms. [file 13287_2020_1993_MOESM2_ESM.tif]
